# Metagenomic next-generation sequencing and conventional microbiology for microbial profiling in biliary tract infections: a comparative study with clinical stratification

**DOI:** 10.3389/fmicb.2026.1799474

**Published:** 2026-03-30

**Authors:** Jianmin Ren, Ziling Lan, Cheng Wang, Jinnuo Zhu, Mei Li, Jianfen Xu, Yiyang Lu, Jianfei Tu, Xiaoyao Zhang, Lidija Boskovic, Jiansheng Huang, Xiaolei Hu

**Affiliations:** 1 Graduate Joint Training Base of Zhejiang Chinese Medical University (Lishui Joint Training Base - Lishui Central Hospital), Lishui, China; 2 Department of Clinical Laboratory, The Fifth Affiliated Hospital of Wenzhou Medical University, Lishui, Zhejiang, China; 3 Department of Microbiology and Genetics, Clinical Hospital Center Zvezdara, Belgrade, Serbia

**Keywords:** anaerobic bacteria, biliary microbiome, biliary tract infection, clinical microbiology, metagenomic next-generation sequencing, polymicrobial infection

## Abstract

**Background:**

Biliary tract infections (BTIs) are clinically important conditions characterized by complex heterogeneous microbial profiles. However, the complete spectrum of implicated microorganisms, particularly anaerobic species and polymicrobial signatures, remains insufficiently defined due to the inherent limitations of conventional microbiological methods.

**Methods:**

In this retrospective study, bile samples obtained from patients with BTIs were analyzed using DNA- and RNA-based metagenomic next-generation sequencing (mNGS) alongside conventional microbiological techniques, including culture and smear microscopy. The breadth of microbial detection across these methods was systematically compared. Microbial profiles were further stratified according to clinical variables, including sex, infection site, and disease severity.

**Results:**

Metagenomic sequencing revealed a broad array of microorganisms, including bacteria, fungi, viruses, and parasites, with bacteria constituting the predominant component. A considerable proportion of identified bacterial taxa were obligate or facultative anaerobes, and polymicrobial detection patterns were frequently observed. Gram-positive genera such as *Streptococcus* and *Enterococcus*, as well as Gram-negative genera including *Klebsiella* and *Escherichia*, were commonly detected. Male patients had higher detection rates of *Enterococcus* and *Enterobacter*, whereas *Staphylococcus* species were more frequently identified in gallbladder infections than in bile duct infections. In this cohort, DNA-based mNGS showed broader microbial detection coverage than RNA-based mNGS and conventional culture methods.

**Conclusion:**

These results underscore the complexity of microbial detection in bile-associated infections and support the value of metagenomic sequencing for comprehensive microbial characterization in BTIs. The marked heterogeneity in microbial distribution indicates that bile samples from patients with BTIs commonly show polymicrobial profiles rather than a uniform single-organism profile.

## 1 Introduction

Biliary tract infections (BTIs), primarily including cholecystitis and cholangitis, are common clinical conditions that typically arise secondary to biliary calculi or ductal obstruction. In severe cases, these infections may progress to sepsis or multiple organ failure, resulting in life-threatening consequences. Previous studies have reported that more than 90% of patients with acute cholangitis have positive cultures from bile, biliary stones, or obstructed stents, with polymicrobial infections predominated by Gram-negative and Gram-positive enteric organisms (Zhang et al., 2023; van den Hazel et al., 1994). Anaerobic bacteria, such as *Bacteroides* and *Clostridium* species, are also frequently implicated.

Despite their clinical importance, routine bile culture practices in many centers are largely confined to aerobic conditions and therefore often fail to detect anaerobic, fastidious, or atypical pathogens, including viruses and parasites. Therefore, the true microbial diversity associated with BTIs is likely underestimated. Conventional culture techniques are further limited by prolonged turnaround times, reduced sensitivity following previous antibiotic exposure, and incomplete pathogen identification, all of which may delay the initiation of targeted antimicrobial therapy. Moreover, recent evidence indicates that pathogen composition varies substantially across different types and etiologies of BTIs, underscoring the need for more comprehensive and sensitive diagnostic approaches (Gassiep et al., 2024; Hao et al., 2023).

Alongside traditional culture-based investigations, sequencing-based studies have begun to broaden understanding of the biliary microbiome across various biliary disease contexts. For example, 16S rRNA gene sequencing has shown that microbial composition and diversity may differ by stone location (e.g., bile duct versus gallbladder), supporting the concept of site-specific heterogeneity within the biliary tract (Park and Park, 2024). More recent bile-focused sequencing studies in broader cohorts with biliary obstruction have reported higher pathogen detection rates than routine culture and have suggested disease-dependent differences in microbial profiles. However, many of these investigations were largely descriptive and relied on amplicon-based methods rather than clinical metagenomic next-generation sequencing (mNGS) workflows (Wang et al., 2025).

At the same time, the existing literature remains heterogeneous with respect to sample type (bile, biliary stents, or mixed procedural specimens), sequencing strategy (16S/ITS amplicon sequencing versus metagenomic approaches), and clinical focus (e.g., gallstones, biliary obstruction, or device-related biofilm) (Cacaci et al., 2024). Previous comparative studies have also suggested that sequencing-based methods have greater diagnostic value than conventional culture for biliary infections (Dyrhovden et al., 2020). However, the direct application of current biliary microbiome findings to routine microbiological decision-making in BTIs remains limited. Therefore, this study was designed to evaluate bile-based mNGS in a clinically well-defined BTI cohort and to correlate microbial detection profiles with clinically relevant stratification variables within a real-world diagnostic framework.

Over the past decade, mNGS has emerged as a culture-independent, high-throughput technology capable of unbiased detection of bacteria, viruses, fungi, and parasites directly from clinical specimens (Torres Montaguth et al., 2026). Since approximately 2015–2016, mNGS has been increasingly implemented in both research and clinical settings, particularly for central nervous system and severe respiratory infections, where conventional diagnostic methods often demonstrate limited sensitivity. Accumulating evidence suggests that mNGS can substantially enhance pathogen detection rates and shorten diagnostic timelines, providing valuable adjunctive information to support precision antimicrobial therapy.

However, the application of mNGS in BTIs remains relatively underexplored. Most published studies have focused on blood, cerebrospinal fluid, or respiratory specimens, whereas systematic analyses of bile samples are comparatively scarce. Existing reports on biliary mNGS are generally small-scale or primarily descriptive, with limited evaluation of pathogen distribution across different etiologies and severity grades, and insufficient correlation with clinical parameters. These gaps have impeded the integration of mNGS from exploratory research to routine clinical decision-making in BTIs.

In this context, this study applies mNGS to bile samples obtained from patients with BTIs of diverse etiologies and severity levels. Through comprehensive characterization of the biliary microbial landscape and systematic correlation of pathogen distribution with clinical features and disease severity, this study seeks to clarify the diagnostic value of mNGS in BTIs and to assess its potential role in providing supplementary microbiological evidence to inform antimicrobial decision-making. The findings aim to support the rational integration of mNGS into the clinical management of biliary tract infections.

It should be emphasized that mNGS provides microbiological information by identifying potential pathogens and does not constitute a standalone diagnostic or therapeutic modality.

## 2 Materials and methods

### 2.1 Study population and ethical considerations

A total of 100 consecutive bile specimens from patients admitted to Lishui Central Hospital between October 2021 and May 2022 were included in the study dataset, based on the availability of residual bile samples and corresponding clinical records. The primary analytic cohort comprised 99 patients who met the predefined diagnostic criteria for BTI. One additional case of non-infectious gallbladder carcinoma was retained solely as a reference and was excluded from all BTI subgroup analyses.

Patients in the BTI cohort were diagnosed according to established clinical criteria and underwent either ultrasound-guided digital subtraction angiography (DSA)-assisted percutaneous transhepatic biliary drainage (PTBD) (*n* = 48) or surgical cholecystectomy (*n* = 51). In accordance with routine clinical management of suspected BTIs, some patients received empirical antibiotic therapy before bile sampling and invasive intervention. As this investigation was retrospective and based on routine clinical practice, detailed information regarding pre-sampling antibiotic exposure, including timing, duration, and specific regimens, was not consistently available and was therefore not incorporated as a prespecified stratification variable. Bile specimens were collected aseptically during the aforementioned procedures. The samples analyzed in this study comprised residual material remaining after completion of routine clinical microbiological testing.

The study protocol was reviewed and approved by the Institutional Ethics Committee of the Fifth Affiliated Hospital of Wenzhou Medical University. Owing to the retrospective design and the use of discarded clinical specimens with fully anonymized data, the requirement for informed consent was waived. The investigators had no access to identifiable personal information.

### 2.2 Inclusion and exclusion criteria

*Inclusion criteria*

- A diagnosis of acute cholecystitis or acute cholangitis in accordance with the Diagnosis and Treatment Guidelines for Acute Biliary System Infections (2021 Edition) (Biliary Surgery Group of the Chinese Society of Surgery, 2021).
- Completion of standard informed consent procedures for invasive clinical interventions, such as percutaneous transhepatic biliary drainage (PTBD) or cholecystectomy.
- Availability of residual bile specimens and corresponding clinical data suitable for retrospective analysis.

*Exclusion Criteria*

- Evidence of an active infection originating from a non-biliary source.
- Bile specimens with insufficient volume, improper storage conditions, or suspected contamination during collection or transport.
- Incomplete or missing clinical data.

All BTI-focused statistical comparisons and clinical subgroup analyses were performed in the BTI analytic cohort (*n* = 99), unless otherwise specified.

### 2.3 Patient grouping and classification

Disease severity was classified according to the Tokyo Guidelines 2018 (TG18) (Okamoto et al., 2018), which stratifies acute cholangitis and cholecystitis into Grade I (mild), Grade II (moderate), and Grade III (severe). All patients in the BTI analytic cohort met the diagnostic criteria for acute biliary tract infection, consistent with the ICD-11 definitions of acute cholecystitis and acute cholangitis. Patients categorized as Grade II or Grade III underwent urgent PTBD under ultrasound and DSA guidance. For subgroup analyses, Grade II and Grade III cases were combined into a moderate-to-severe group as both categories typically required urgent intervention at our center and the number of Grade III cases was limited. In comparison, patients with Grade I disease were initially managed conservatively with antibiotic therapy and/or clinical observation, followed by elective cholecystectomy performed 2–4 months later.

Based on the primary site of infection, patients were classified into cholecystitis and cholangitis groups. Further stratification incorporated underlying etiologies, imaging findings, and clinical features, including malignant obstructive cholangitis, cholecystolithiasis with inflammation, cholangiolithiasis with inflammation, acalculous cholecystitis, and cholesterol polyps with associated inflammation.

One non-BTI case of non-infectious gallbladder carcinoma was retained solely as a reference and was excluded from all BTI subgroup analyses.

### 2.4 Bacterial smear and gram staining

A 0.5 mL aliquot of each bile specimen was subjected to cytocentrifugation to generate cytospin slides, which were subsequently stained using the Gram method. The stained preparations were examined under light microscopy at ×1,000 oil-immersion magnification to assess bacterial morphology (cocci, bacilli, or mixed forms), Gram-staining characteristics (Gram-positive or Gram-negative), and cellular arrangement (e.g., chains or clusters). Two experienced microscopists independently evaluated all slides, and any discordant interpretations were adjudicated by a third senior examiner.

### 2.5 Conventional bacterial culture

A 200 μL aliquot of each bile specimen was inoculated onto blood agar plates using standard streaking techniques and incubated at 35 °C for 48 h in a 5–10% CO₂ atmosphere. Plates showing no visible growth at 48 h were further incubated for up to 72 h. Conventional culture was conducted exclusively under aerobic conditions, and routine anaerobic culture was not systematically performed.

All morphologically distinct colonies observed on the primary culture plates were individually selected and subcultured onto fresh blood agar plates to obtain pure isolates. Species identification was carried out using the Vitek MS matrix-assisted laser desorption/ionization time-of-flight (MALDI-TOF) mass spectrometry system (bioMérieux) in accordance with the manufacturer’s instructions. Identification confidence values ≥99% were regarded as reliable. In cases of polymicrobial growth, all recovered bacterial species were comprehensively identified and reported. Relative predominance was determined based on colony abundance on the primary culture plates. Specimens demonstrating no visible growth after 72 h of incubation were reported as “no cultivable bacteria detected.”

Results obtained from conventional culture served as the reference standard for comparison with molecular diagnostic approaches.

### 2.6 Metagenomic next-generation sequencing and data analysis

Bile specimens were promptly stored at −80 °C following collection and preserved under controlled conditions until nucleic acid extraction to minimize degradation and contamination. Moreover, mNGS was performed by Guangzhou Weiyuan Gene Technology Co., Ltd. using its standardized clinical mNGS workflow established in 2022.

Total nucleic acids were extracted from each bile sample, with DNA and RNA processed separately. DNA was isolated using an automated magnetic bead–based extraction system, and RNA was purified using column-based extraction kits according to the manufacturers’ protocols. The extracted RNA was reverse-transcribed into complementary DNA (cDNA) before library preparation. Negative controls were included during nucleic acid extraction to monitor potential contamination. Sequencing libraries were constructed using standard metagenomic procedures, including nucleic acid fragmentation, end repair, adapter ligation, and library amplification. DNA libraries and RNA-derived cDNA libraries were prepared independently. High-throughput sequencing was conducted on the BGI MGISEQ-200 platform using a short-read shotgun metagenomic sequencing strategy (read length approximately 100–150 bp).

In this cohort, all bile specimens underwent both DNA- and RNA-based library preparation within the same clinical testing workflow. This approach was retained because the causative pathogens were unknown at the time of testing and could include bacteria, fungi, DNA viruses, or RNA viruses. The RNA-derived library was analyzed in parallel to preserve detection scope for potential RNA pathogens. In the present study, DNA- and RNA-based outputs were recorded and compared as separate reportable results generated under the same workflow.

Raw sequencing data were analyzed using the proprietary bioinformatics pipeline provided by the sequencing service. The analytical workflow included: (1) quality control to remove low-quality reads and adapter sequences; (2) subtraction of host-derived sequences by alignment to the human reference genome; (3) alignment-based mapping of high-quality, non-host reads to curated microbial reference databases covering bacteria, fungi, viruses, and parasites; and (4) taxonomic classification with automated generation of microbial detection reports.

Microorganisms were reported according to predefined quality-control criteria and read-abundance thresholds specified in the 2022 standardized protocol, to reduce background noise and false-positive signals, particularly in low-biomass specimens such as bile. Final mNGS reports provided qualitative and semi-quantitative data on detected microorganisms. Under the routine reporting framework, different interpretation thresholds were applied by pathogen category. This was an untargeted short-read shotgun metagenomic workflow rather than a predefined organism-specific target panel; therefore, reportability was determined by category-specific reporting rules rather than by a fixed number of organism-specific targets.

For bacteria (excluding *Mycobacterium* spp.), fungi (excluding *Cryptococcus* spp.), and parasites, a microorganism was considered reportable when its coverage ranked within the top 10 among organisms of the same category and it was absent in the no-template control (NTC); if the organism was also detected in the NTC, it was retained only when the ratio of reads per million between the clinical sample and the NTC (RPM_sample_/RPM_NTC_) was >10. Here, coverage refers to the platform-reported coverage metric used for within-category ranking. For viruses, *Mycobacterium tuberculosis*, and *Cryptococcus* spp., a microorganism was considered reportable when at least one unique read was mapped at the species level and the organism was absent in the NTC; if it was also detected in the NTC, it was retained only when RPM_sample_/RPM_NTC_ was >5. Here, unique read was defined according to the platform’s reporting output at the species level. In this study, presence/positivity referred to a taxon retained as reportable in the finalized clinical mNGS report, whereas absence/negativity referred to a taxon not retained as reportable after quality-control and background-filtering procedures.

For bacterial taxa, results were primarily summarized and analyzed at the genus level to enhance statistical robustness, given the limited discriminatory resolution of short-read metagenomic sequencing for closely related species. Genus-level data were therefore used for formal statistical comparisons, while clinically relevant taxa reported at the species or species-complex level were summarized descriptively in Supplementary Table S1. For these descriptively reported taxa, relative abundance (%) and read counts were expressed as median [IQR] across positive samples when the designation did not represent a mixed-species assignment. Species-level identification was retained when sequence data permitted confident discrimination. Fungi, viruses, and parasites were reported at the species level.

This investigation was a retrospective analysis based on finalized clinical mNGS reports. Putative background or contaminant signals were handled primarily by relative-to-NTC filtering and were interpreted in conjunction with a platform-derived background library generated from blank controls. Taxa deemed consistent with the background signal or likely contamination were not reported as positive findings. No raw-data reanalysis or additional *post hoc* research thresholds were applied.

Typical clinical mNGS performed on MGISEQ platforms generates tens of millions of raw reads per library, providing adequate sequencing depth for qualitative microbial detection. Although per-sample read counts were recorded and summarized descriptively (Supplementary Table S1), sequencing depth was not incorporated into downstream statistical analyses, and no abundance-based subgroup modeling was undertaken.

### 2.7 Statistical analysis

All statistical analyses were conducted using SPSS software (version 26.0; IBM Corp.). Normality of continuous variables was assessed using the Shapiro–Wilk test. Variables following a normal distribution are presented as mean ± standard deviation, whereas non-normally distributed data are expressed as median with interquartile range (IQR). Between-group comparisons of non-normally distributed continuous variables were performed using the Mann–Whitney U test.

Categorical variables are summarized as counts and percentages. Associations between pathogen detection and clinical parameters, including sex, age group, disease severity, infection site, and the presence of stones or tumors, were evaluated by comparing detection frequencies across subgroups using the Chi-square test or Fisher’s exact test, as appropriate. All tests were two-sided, and *p*-values < 0.05 were considered statistically significant. To account for multiple testing, the false discovery rate (FDR) was controlled using the Benjamini–Hochberg method, and adjusted *p*-values are reported as *q*-values.

Subgroup analyses were considered exploratory and descriptive. Given the limited sample size within certain strata and the large number of taxon-level comparisons, multivariable modeling was not undertaken, and subgroup findings were interpreted with caution. Unless otherwise specified, statistical analyses and subgroup comparisons were confined to the BTI analytic cohort (*n* = 99). Pre-sampling antibiotic exposure was not included as a covariate in the primary subgroup analyses because detailed exposure data were not consistently available from routine medical records.

## 3 Results

### 3.1 Patient characteristics and demographics

**Figure 1:**
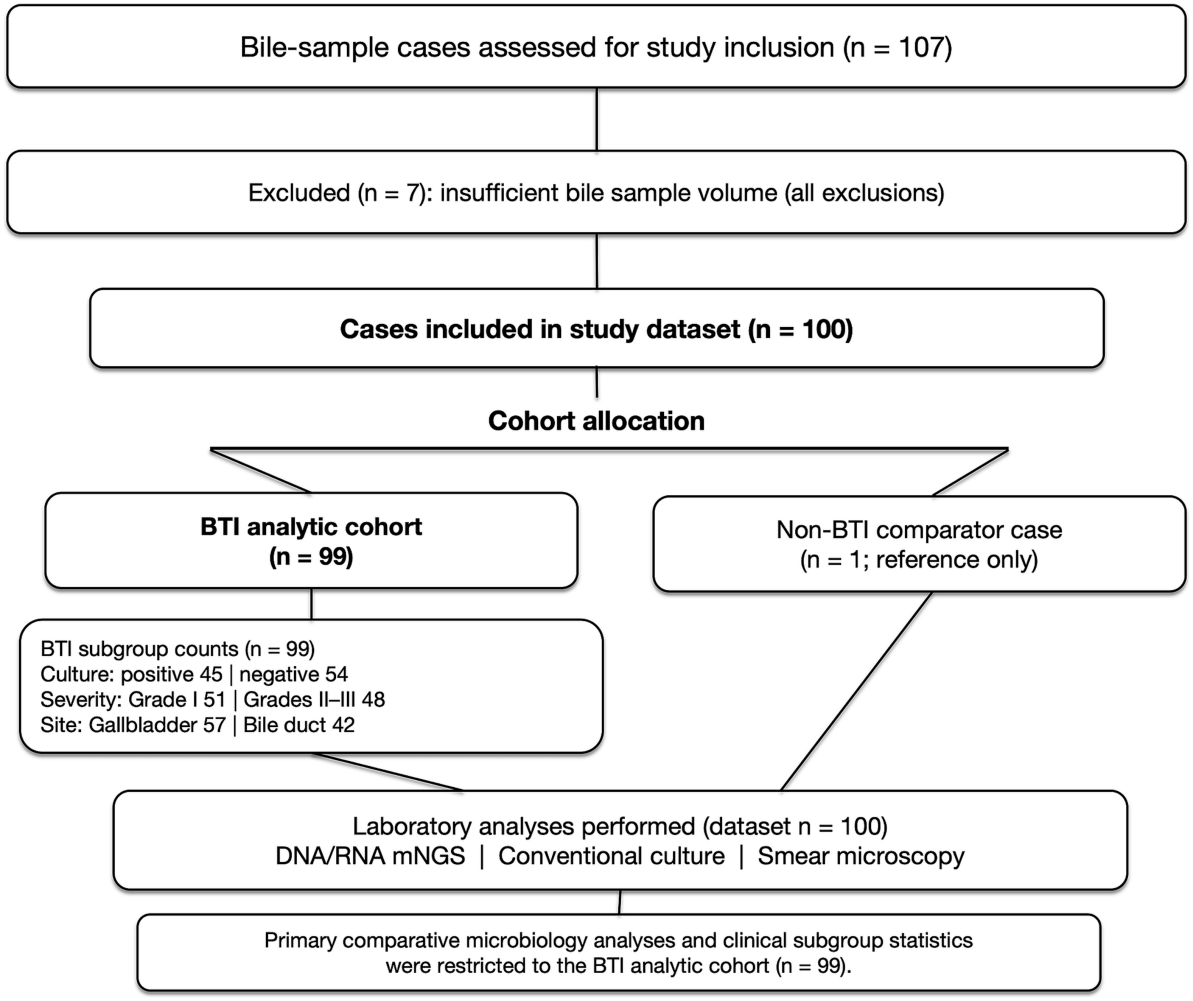
Study flowchart and derivation of analytical subgroups. Bile specimens collected during the study period were screened for eligibility. After excluding cases with insufficient sample volume, 100 cases were included in the final study dataset. The primary analytic cohort consisted of 99 patients who met the predefined diagnostic criteria for biliary tract infection (BTI). One non-BTI case (non-infectious gallbladder carcinoma) was retained solely as a reference and excluded from all BTI subgroup analyses. All included cases underwent DNA- and RNA-based metagenomic next-generation sequencing (mNGS), conventional culture, and smear microscopy. Comparative microbiological assessments and clinical subgroup analyses were confined to the BTI analytic cohort (*n* = 99).

A total of 100 bile-sample cases were included in the study dataset. Among these, 99 cases fulfilled the predefined diagnostic criteria for BTI and comprised the primary analytic cohort. One non-BTI case (non-infectious gallbladder carcinoma) was retained solely as a reference and excluded from comparative and subgroup analyses. All comparative microbiological evaluations and clinical subgroup analyses were confined to the BTI analytic cohort (Figure 1). Within the BTI cohort, 55 patients were male, and 44 were female. Male patients were significantly older than female patients (*p* < 0.001). Baseline demographic and clinical characteristics of the cohort are presented in Table 1. The etiological distribution included 35 cases of malignant obstructive cholangitis, 43 cases of cholecystitis with gallstones, 7 cases of cholangitis with gallstones, 7 cases of acalculous cholecystitis, and 7 cases of cholesterol polyps with associated inflammation.

**Table 1:** Baseline characteristics of the BTI analytic cohort (*n* = 99).

Characteristic	*n* (%)	Remarks/subgroup details
Total patients	99	
Sex		
Male	55 (55.6)	Mean age: 64.3 ± 14.5 years
Female	44 (44.4)	Mean age: 53.7 ± 14.1 years
Diagnosis		
Malignant obstructive cholangitis	35 (35.4)	
Cholecystolithiasis with inflammation	43 (43.4)	
Cholangitis with gallstones	7 (7.1)	
Acalculous cholecystitis	7 (7.1)	
Cholesterol polyps with inflammation	7 (7.1)	
Severity group (TG18/TG13)		
Grade I (Mild)—surgery group	51 (51.5)	Mean age: 51.8 ± 14.3 years
Male	21	Mean age: 56.2 ± 14.8 years
Female	30	Mean age: 49.2 ± 13.4 years
Grade II and III (Moderate–Severe)—PTBD group	48 (48.5)	Mean age: 67.0 ± 10.6 years
Grade II (Moderate)	35 (35.4)	
Grade III (Severe)	13 (13.1)	
Male	35	Mean age: 68.5 ± 11.2 years
Female	13	Mean age: 60.8 ± 11.4 years

SD, standard deviation.

Age comparisons were performed using the Mann–Whitney U test. A statistically significant age difference was identified between male and female patients across the overall cohort (*P* < 0.001). Similarly, age differed significantly between the surgery (Grade I) group and the PTBD (Grade II/III) group (*P* < 0.001).

According to the TG18/TG13 severity grading system, patients were categorized into a mild group (*n* = 51) managed with cholecystectomy and a moderate-to-severe group (*n* = 48) treated with PTBD. Detailed demographic characteristics, clinical features, and key microbiological findings for these groups are summarized in Table 1. Patients in the moderate-to-severe (PTBD) group were significantly older (mean age 67.0 ± 10.6 years) than those in the mild (surgery) group (mean age 51.8 ± 14.3 years) (*p* < 0.001), indicating that the PTBD cohort was predominantly older. The PTBD group had a higher proportion of male patients, whereas the mild surgical group was comparatively younger and included a greater proportion of female patients.

### 3.2 Overall composition of the biliary microbial detection profiles

Among all mNGS-positive bile specimens, complex microbial detection patterns were observed, predominantly composed of bacterial taxa and frequently characterized by polymicrobial co-detection. Both Gram-positive and Gram-negative organisms were commonly identified, with obligate and facultative anaerobic genera representing a substantial proportion of the detected bacteria. In addition to bacterial taxa, fungal and viral organisms were identified in a subset of samples, indicating that the detected microbial landscape comprised a broad spectrum of microorganisms rather than isolated single-pathogen findings.

### 3.3 Impact of detection method on microorganism identification in biliary tract infections

#### 3.3.1 Comparison of DNA- and RNA-based mNGS detection in bile samples

Because the causative pathogens were unknown at the time of testing and could include RNA pathogens, both DNA- and RNA-based mNGS were performed on all 100 bile specimens, and their respective outputs were compared within this cohort. The positive detection rate for DNA sequencing was 73%, significantly exceeding that of RNA sequencing (51%) (*p* < 0.01). Concordant Microbial profiles were identified by both methods in 53 samples (53%). In the remaining 47 samples, DNA-based mNGS demonstrated a broader detection spectrum, identifying additional taxa not captured by RNA sequencing (Table 2).

**Table 2:** Microorganisms detected in bile samples by DNA-based mNGS, RNA-based mNGS, and conventional culture.

Phylum/kingdom	Taxon (genus or species)	mNGS (*n*)		Culture (*n*)
		DNA	RNA	
Bacillota	*Streptococcus*	23	10	11
	*Lactococcus*	2	1	1
	*Enterococcus*	22	10	16
	*Granulicatella*	5	4	–
	*Abiotrophia*	3	2	–
	*Staphylococcus*	13	3	5
	*Gemella*	2	1	–
	*Cohnella*	2	0	–
	*Lactobacillus*	2	1	1
	*Leuconostoc*	1	1	–
	Peptoniphilus*	1	0	–
	*Clostridium*	6	2	1
	*Fusobacterium*	7	5	1
	Hungatella*	2	1	–
	*Micromonospora*	7	4	1
	*Selenomonas*	2	1	–
	Catonella*	1	1	–
	*Pseudoramibacter*	1	0	–
	*Veillonella*	8	4	1
	*Weissella*	1	1	–
Proteobacteria	*Klebsiella*	23	9	14
	*Proteus*	3	1	–
	*Citrobacter*	2	0	1
	*Escherichia*	18	12	9
	*Enterobacter*	11	6	7
	*Salmonella*	1	1	1
	*Morganella*	3	1	1
	*Serratia*	1	0	–
	*Haemophilus*	7	2	1
	*Pseudomonas*	2	1	–
	*Acinetobacter*	1	0	1
	*Shewanella*	3	1	2
	*Stenotrophomonas*	1	1	–
	*Campylobacter*	9	4	–
	*Helicobacter*	1	0	–
	*Eggerthella*	3	2	–
	*Burkholderia*	1	0	–
Actinobacteria	*Actinomyces*	11	5	–
	*Rothia*	4	2	–
	*Propionibacterium*	3	1	–
	*Atopobium*	2	2	–
	*Olsenella*	2	1	–
	Bifidobacterium*	1	1	–
Bacteroidota	Bacteroides*	6	3	–
	Dysgonomonas*	1	1	–
	*Porphyromonas*	3	0	–
	Prevotella*	1	0	–
	Tannerella*	1	0	–
Verrucomicrobia	Akkermansia*	1	1	–
Fungi	*Candida albicans*	6	4	5
	*Candida tropicalis*	1	0	1
	*Candida glabrata*	2	2	1
	*Candida parapsilosis*	3	3	2
	*Fusarium*	1	0	–
	*Aspergillus fumigatus*	2	0	–
Virus	*Torque teno virus (TTV)*	6	2	–
	*Cytomegalovirus (CMV)*	4	0	–
	*Epstein–Barr virus (EBV)*	3	0	–
	*Human papillomavirus type 33 (HPV-33)*	0	1	–
	*Human herpesvirus 7 (HHV-7)*	1	0	–
	*Hepatitis B virus (HBV)*	4	6	–
Parasite	*Clonorchis sinensis*	1	0	–

* Indicates obligate or commonly anaerobic genera.

“–” Indicates that no microorganisms were detected by the corresponding method.

Several common biliary bacterial genera were not detected by RNA sequencing, including *Klebsiella* (14 cases), *Streptococcus* (13 cases), *Enterococcus* (12 cases), *Staphylococcus* (10 cases), and *Escherichia* (6 cases). Under the conditions used in this cohort, RNA-based mNGS provided lower bacterial detection coverage than DNA-based mNGS. These differences should be interpreted as workflow-dependent differences in reportable detection rather than as evidence for or against active infection. Therefore, all subsequent comparative analyses were conducted using DNA-based mNGS data. For statistical and comparative purposes, bacterial taxa were primarily analyzed at the genus level, whereas fungi, viruses, and parasites were evaluated at the species level.

Bacterial taxa were summarized at the genus level for formal statistical analyses. Clinically relevant bacterial taxa reported at the species or species-complex level are presented descriptively in Supplementary Table S1. Fungi, viruses, and parasites are reported at the species level.

#### 3.3.2 Comparative diagnostic performance of mNGS, conventional culture, and smear microscopy

A comparative evaluation of microbial detection was conducted across 100 bile specimens using mNGS, conventional culture, and smear microscopy performed in parallel. The overall detection rate of mNGS (73%, 73/100) was significantly higher than that of conventional culture (45%, 45/100) and smear microscopy (40%, 40/100) (both *p* < 0.01). At the individual-specimen level, mNGS identified microorganisms in 17 cases, whereas conventional culture and smear microscopy yielded unique positive findings in only 4 and 2 cases, respectively (Table 2). These results indicate that, within the routine diagnostic workflow used in this cohort, mNGS yielded a higher microbial detection rate than conventional culture and smear microscopy.

mNGS detected a broad range of microorganisms, encompassing 64 genera, including aerobic and anaerobic bacteria, fungi, viruses, and parasites. The most frequently identified bacterial genera were *Streptococcus* (23%), *Klebsiella* (23%), *Enterococcus* (22%), *Escherichia* (18%), *Staphylococcus* (13%), and *Enterobacter* (11%). A total of 70 mNGS detections of obligate or facultative anaerobic bacteria representing 32 genera, were identified, substantially exceeding the four anaerobic strains recovered by culture. Furthermore, mNGS detected nucleic acid signals for six fungal species (12 cases), six viral species (18 cases), and one parasitic species (Table 2).

Clinically relevant bacterial species or species-complex taxon labels identified by mNGS are summarized descriptively in Supplementary Table S1, along with sample-level detection counts and semi-quantitative indicators (relative abundance and read counts, expressed as median [IQR]). These results represent nucleic acid detections reported in the literature. They should be interpreted in the context of clinical findings, particularly when viral signals or low-abundance organisms are detected in bile.

#### 3.3.3 Detection of anaerobic bacteria and polymicrobial infections by mNGS

Among the 73 patients with positive mNGS results, anaerobic bacteria were identified in 23 cases (31.5%), totaling 70 strains. The most frequently reported anaerobic genus was *Actinomyces* (11.4%, 8/70), followed by *Fusobacterium* (8.6%, 6/70), *Parvimonas* (8.6%, 6/70), and *Veillonella* (8.6%, 6/70) (Table 2). Selected anaerobic taxa reported at the species or species-complex level are presented descriptively in Supplementary Table S1.

**Figure 2:**
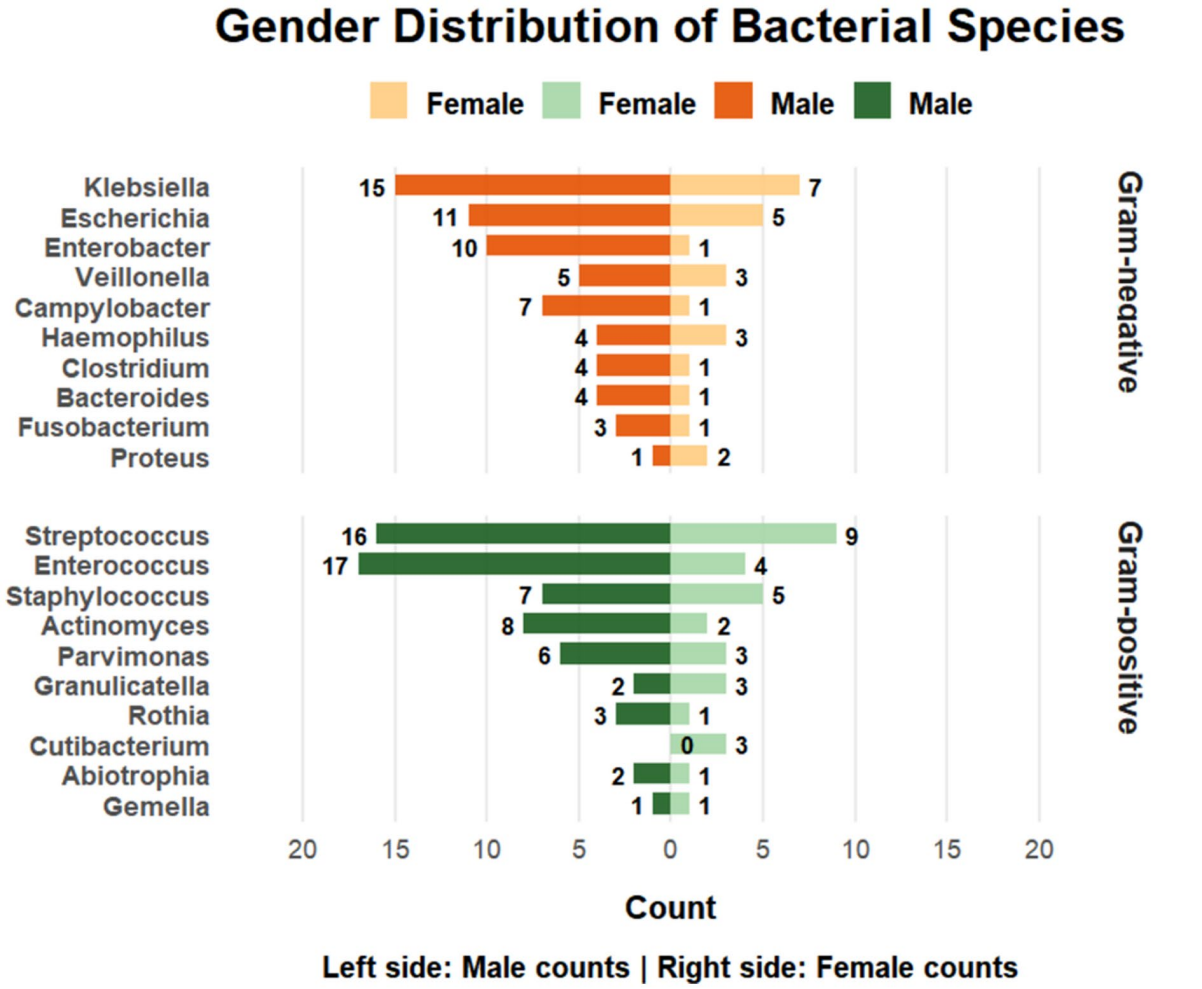
Association between gender and microbial detection in bile samples. Male subjects showed a significantly enriched prevalence of *Enterococcus* spp. (31.5% vs. 8.9%) and *Enterobacter* spp. (18.5% vs. 2.2%) compared to females (*q* < 0.05). Figures were generated using GraphPad Prism and Adobe Illustrator.

**Figure 3:**
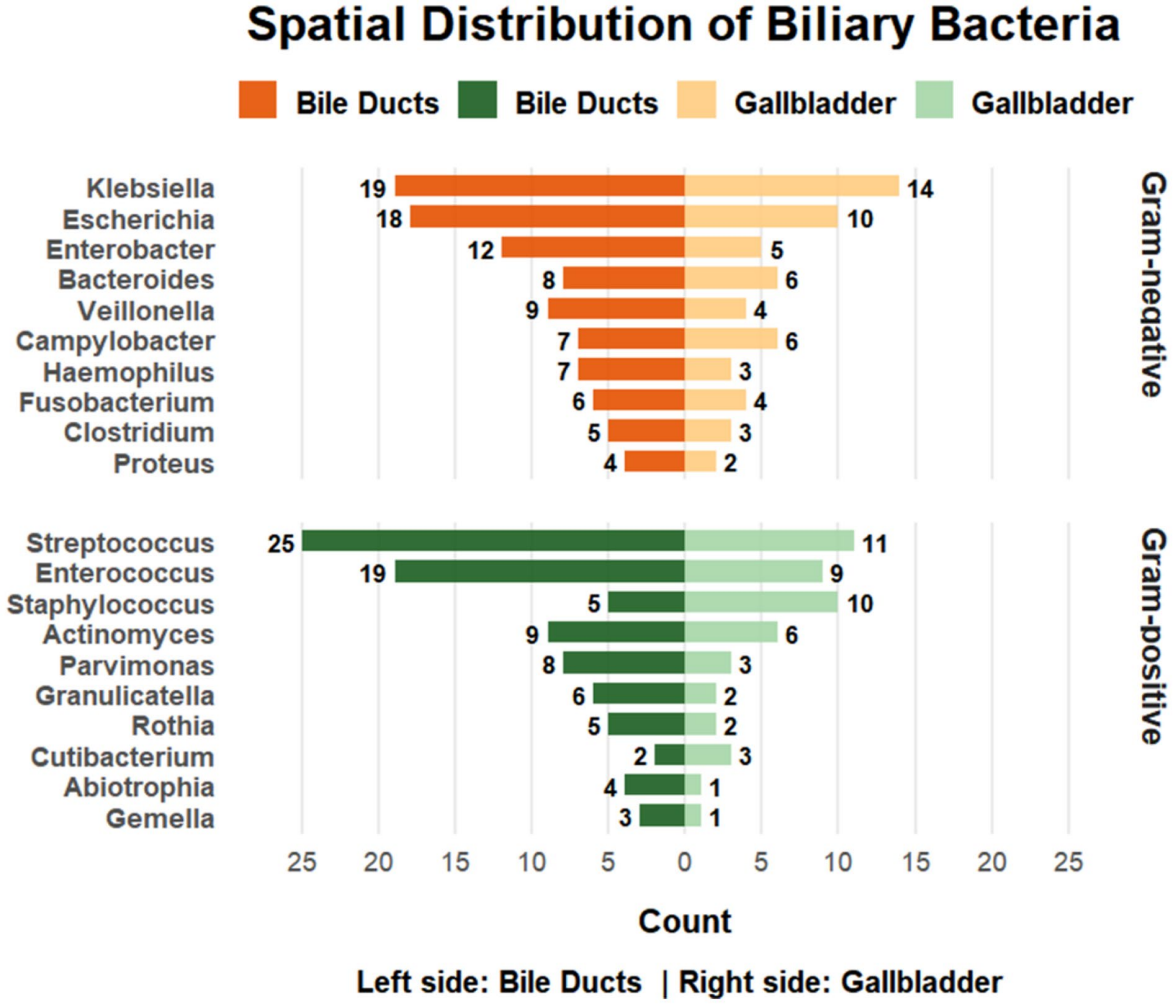
Differences in microbial detection between gallbladder and bile duct samples. In this exploratory site-based comparison, *Staphylococcus* spp. showed a higher detection frequency in gallbladder than in bile duct samples (17.5% vs. 11.9%, *q* < 0.05); this signal should be interpreted cautiously in the context of multiple subgroup comparisons.

Further evaluation of these 73 mNGS-positive samples demonstrated a high prevalence of polymicrobial detection, defined as the identification of two or more microorganisms, which was observed in 80.8% (59/73) of cases. In comparison, single-pathogen detection occurred in only 19.2% (14/73) of cases (Table 3). These results indicate that, under the routine predominantly aerobic culture workflow applied in this cohort, mNGS identified a broader spectrum of microorganisms, including anaerobic taxa and polymicrobial patterns, than conventional culture (Figures 2, 3).

**Table 3:** Microbial profile detected by mNGS in patients with biliary tract infections.

Detection category	*n* (%)
Culture-positive samples	45 (45.0)
Smear-positive samples	40 (40.0)
mNGS-positive samples	73 (73.0)
Bacteria detected by mNGS	68 (93.2)
Fungi detected by mNGS	12 (16.4)
Viruses detected by mNGS	18 (24.7)
Parasites detected by mNGS	1 (1.4)
Anaerobic bacteria detected by mNGS	23 (31.5)
Single-pathogen infections by mNGS	14 (19.2)
Polymicrobial infections by mNGS	59 (80.8)

Percentages for bacteria, fungi, viruses, parasites, anaerobic bacteria, and infection types are calculated out of the 73 mNGS-positive samples.

### 3.4 Analysis of microbial profiles across clinical subgroups

#### 3.4.1 Association of microbial distribution with patient gender

Among the 99 biliary tract infection specimens, microorganisms were detected in 54 male patients (77.8%) and 45 female patients (66.7%), yielding a total of 217 and 76 detected microorganisms, respectively. In this exploratory subgroup analysis, male patients demonstrated higher observed detection rates across the three principal microbial categories, Gram-positive bacteria, Gram-negative bacteria, and fungi/viruses (all *q* < 0.01).

At the genus level, *Enterococcus* was more frequently identified in males (31.5%, 17/54) than in females (8.9%, 4/45), and *Enterobacter* was similarly detected more often in males (18.5%, 10/54) compared with females (2.2%, 1/45) (both *q* < 0.05). Given the relatively small subgroup sizes and the multiple comparisons performed, these results should be regarded as exploratory rather than definitive epidemiological patterns. No additional genus-level differences met the prespecified false discovery rate threshold (Supplementary Table S1).

#### 3.4.2 Impact of age on microbial distribution

According to the WHO age classification criteria, patients were stratified into an elderly group (≥60 years, *n* = 52) and a younger group (18–59 years, *n* = 47). Microorganisms were detected in 76.9% of elderly patients (211 strains) and 72.3% of the younger patients (171 strains). After adjustment for multiple testing using the Benjamini–Hochberg procedure, no statistically significant differences in the distribution of individual microbial genera were identified between the two age groups (all *q*-values ≥ 0.05).

#### 3.4.3 Correlation between disease severity and microbial profiles

The moderate-to-severe (PTBD) group accounted for 313 detected microorganisms, whereas in the mild (surgery) group accounted for 204. Although the absolute number of detected microorganisms was greater in the moderate-to-severe group, the overall genus-level distribution patterns were comparable between the two groups. After adjustment for multiple comparisons using FDR correction, no statistically significant differences were identified for any individual genus (all *q* > 0.05), suggesting no evident association between disease severity and genus-level microbial detection rates in this cohort.

#### 3.4.4 Microbial distribution by infection site

In this exploratory analysis stratified by infection site, Staphylococcus was detected more frequently in gallbladder specimens (17.5%, 10/57) than in bile duct infections (11.9%, 5/42) (*q* < 0.05). Detection rates for all other microbial genera were comparable between the two sites (all *q* ≥ 0.05). These site-based comparisons are descriptive in nature and should be interpreted with caution. The observed association represents an exploratory subgroup signal and was not adjusted for potential confounding factors.

#### 3.4.5 Microbial profiles in patients with and without tumors

The overall number of detected microorganisms and the relative distribution of major microbial genera were comparable between patients with biliary tumors and those without. Following adjustment for multiple comparisons, no statistically significant differences in genus-level detection rates were identified (all *q* ≥ 0.05).

#### 3.4.6 Microbial profiles in patients with and without gallstones

The total number of detected microorganisms was greater in the non-stone group (343 strains) than in the stone group (224 strains). Although the detection rate of Streptococcus showed a nominally significant difference before adjustment for multiple comparisons (*p* = 0.002), this association did not remain statistically significant after FDR correction (*q* = 0.056). No other microorganisms showed significant differences in detection rates between the two groups (all *q* ≥ 0.05).

### 3.5 Characteristic bacterial co-aggregation patterns

mNGS profiling demonstrated recurrent patterns of bacterial co-detection in bile specimens. *Streptococcus*, *Veillonella*, and *Actinomyces* frequently co-occurred. All samples positive for *Veillonella* (8 cases) or *Actinomyces* (11 cases) were concurrently positive for *Streptococcus*. Moreover, all three genera were identified simultaneously detected in six samples, suggesting a potential co-aggregation pattern within the biliary microbial landscape.

## 4 Discussion

Complex, heterogeneous microbial profiles characterized BTIs in this cohort. In this retrospective, report-based analysis, we applied mNGS to define reportable microbial signals in bile specimens and examined variation in detection patterns across clinical subgroups, including sex, age, disease severity, infection site, tumor status, and stone presence. Consistent with previous culture- and sequencing-based investigations, *Escherichia coli* and *Klebsiella pneumoniae* were among the most frequently identified organisms, reinforcing the concept that BTIs are predominantly associated with enteric flora (Li et al., 2019). Compared with routine conventional microbiology, mNGS demonstrated broader microbial detection coverage, particularly for polymicrobial and anaerobic findings. This observation is clinically plausible given the anatomical and microbial interface between the biliary tract and the intestine, as well as the recognized complexity of BTI microbiology. However, these results must be interpreted within the practical diagnostic framework of this study. Conventional microbiology was performed primarily under aerobic conditions, and routine anaerobic culture was not systematically implemented. This methodological imbalance likely reduced recovery of anaerobic and other fastidious organisms by culture, limiting direct equivalence in head-to-head comparison (Sun et al., 2022; Liu et al., 2024). Therefore, these results reflect a broader detection yield under routine clinical workflow rather than unconditional superiority of mNGS across all organism categories.

mNGS also detected fungal, viral, and parasitic nucleic acid signals in bile (Table 3), extending the detectable spectrum beyond that of conventional culture. While these results illustrate the expanded breadth of detectable microbial components, caution is required in their interpretation. As this was a retrospective analysis based on finalized mNGS reports, detected signals represent nucleic acid presence rather than definitive evidence of active infection. This distinction is particularly important for viral or low-abundance findings, where background signal, transient microbial translocation, or clinically irrelevant nucleic acid detection cannot be excluded. Clinical interpretation should therefore be integrated with patient symptoms, laboratory parameters, imaging findings, and procedural context (Xu et al., 2023; Miao et al., 2018).

The observed differences between DNA- and RNA-based mNGS are most appropriately understood as workflow-dependent variations in reportable detection rather than intrinsic differences in diagnostic capability. Factors such as reporting thresholds, background filtering, nucleic acid stability, extraction efficiency, library construction, sequencing depth, and RNA degradation may have contributed to the lower RNA-based detection yield in this cohort. A single total-nucleic-acid library workflow was not evaluated, as all specimens were processed within the routine clinical service workflow, which generated separate DNA- and RNA-based libraries. Under the workflow applied here, DNA-based mNGS provided broader reportable microbial coverage in bile, including common bacterial genera and DNA viral signals.

Anaerobic and polymicrobial detections were prominent features of mNGS-positive bile samples. Given the known limitations of routine anaerobic culture in many clinical settings, this pattern is biologically plausible and consistent with the role of mixed enteric flora in BTIs (Strohäker et al., 2021; Chen et al., 2017; Zhao et al., 2019; Gu et al., 2020; Stathopoulos et al., 2024). In this cohort, anaerobic bacteria were identified in 31.5% (23/73) of mNGS-positive patients, encompassing diverse taxa. These results support the role of sequencing-based methods as complementary tools to conventional microbiology, particularly when polymicrobial infection is suspected or when culture yields are limited.

Exploratory subgroup analyses identified higher genus-level detection frequencies of Enterococcus and Enterobacter in male patients and a higher detection frequency of Staphylococcus in gallbladder compared with bile duct infections. These associations should be interpreted as exploratory observations rather than definitive epidemiological patterns. Although analyses were adjusted for multiple testing using FDR correction, they were descriptive in nature and not designed to establish independent associations. Furthermore, potentially relevant confounders, including pre-sampling antibiotic exposure, timing and type of intervention (e.g., drainage versus surgery), degree of obstruction, and comorbidity burden, were not incorporated into multivariable modeling in this retrospective dataset.

The recurrent co-detection of Streptococcus, Veillonella, and Actinomyces represents a hypothesis-generating observation. This constellation may reflect shared ecological niches or co-aggregation behavior within bile; however, the present data do not permit mechanistic or causal inference. Prospective studies with standardized sampling protocols and comprehensive clinical metadata will be required to determine whether such microbial constellations are reproducible and clinically meaningful.

From a clinical standpoint, mNGS should be regarded as a complementary microbiological modality rather than a standalone diagnostic tool. The diagnosis of BTIs remains a composite clinical determination, and mNGS does not provide phenotypic antimicrobial susceptibility data. Its principal contribution lies in expanding microbiological detection coverage, particularly in polymicrobial and anaerobic contexts, while conventional culture remains indispensable for susceptibility-guided therapeutic decision-making.

Recent reviews addressing microbiota–host interactions in infectious and hepatobiliary contexts offer a broader conceptual framework for interpreting complex microbial detection signals. However, these studies are not specific to BTIs and should not be considered direct comparative evidence for the present cohort (Scarlata et al., 2025; Zeng et al., 2026).

### 4.1 Limitations

This study has several limitations. First, a true non-biliary disease control cohort was not included. In clinical practice, bile sampling is generally performed only during invasive procedures undertaken for specific indications. The single non-BTI case retained in the dataset does not constitute a meaningful disease-versus-control microbiological comparator and was therefore excluded from BTI subgroup analyses. Second, conventional microbiology was conducted primarily under aerobic conditions, and dedicated anaerobic culture was not routinely implemented. This methodological asymmetry likely reduced the recovery of anaerobic and other fastidious organisms by culture. Accordingly, the higher positivity rate observed with mNGS should be interpreted as reflecting broader detection under real-world workflow conditions rather than as a fully balanced comparative estimate of diagnostic performance. Third, empirical antibiotic therapy administered before bile sampling may have influenced culture sensitivity and microbial detection patterns. However, detailed information regarding timing, duration, and specific regimens was not consistently available, precluding standardized adjustment in subgroup analyses. Fourth, although sequencing depth and semi-quantitative mNGS outputs were available, these data were summarized descriptively (relative abundance and read counts, median [IQR]) in Supplementary Table S1. Abundance-based subgroup modeling was not performed.

Finally, this was a retrospective, single-center study with a small sample size, limiting generalizability. Prospective, multicenter investigations incorporating standardized anaerobic culture protocols and more comprehensive covariate collection are warranted.

## 5 Conclusion

In summary, biliary tract infections in this cohort were characterized by complex, heterogeneous microbial profiles, with frequent polymicrobial and anaerobic involvement. Within the context of a predominantly aerobic routine culture workflow, DNA-based metagenomic next-generation sequencing demonstrated broader coverage of reportable microbes than conventional culture, particularly in identifying anaerobic organisms and polymicrobial constellations. Although mNGS does not replace culture-based antimicrobial susceptibility testing, it offers complementary microbiological insight that may improve etiological characterization of biliary tract infections. Prospective, multicenter studies are needed to further define the clinical role and practical value of mNGS-guided diagnostics in the routine management of biliary tract infections.

## Data Availability

The datasets presented in this article are not readily available because they contain clinical patient data subject to privacy and ethical restrictions. Raw datasets are therefore not publicly accessible. De-identified data may be made available to qualified researchers upon reasonable request. Requests should be directed to the corresponding author, Xiaolei Hu. Any data access request will be subject to review by the institutional ethics committee to ensure consistency with the scope of participants’ informed consent, and a formal data-sharing agreement may be required.

## Glossary

BTI: Biliary tract infection
mNGS: Metagenomic next-generation sequencing
NGS: Next-generation sequencing
PTBD: Percutaneous transhepatic biliary drainage
DSA: Digital subtraction angiography
TG18: Tokyo Guidelines 2018
ICD-11: International Classification of Diseases, 11th Revision
MALDI-TOF MS: Matrix-assisted laser desorption/ionization time-of-flight mass spectrometry
DNA: Deoxyribonucleic acid
RNA: Ribonucleic acid
cDNA: Complementary DNA
SD: Standard deviation
IQR: Interquartile range
FDR: False discovery rate
WHO: World Health Organization
HBV: Hepatitis B virus
CMV: Cytomegalovirus
EBV: Epstein–Barr virus
TTV: Torque teno virus
HPV: Human papillomavirus
HHV-7: Human herpesvirus 7
ICU: Intensive care unit
