# Supplementary material for: Metagenomic next-generation sequencing and conventional microbiology for microbial profiling in biliary tract infections: a comparative study with clinical stratification
Source: Front Microbiol. 2026 Mar 30;17:1799474. doi: 10.3389/fmicb.2026.1799474 (PMC13071068; doi:10.3389/fmicb.2026.1799474)
Supplement: Supplementary file 1 [file Table_1.docx]

**Metagenomic Next-Generation Sequencing and Conventional Microbiology for Microbial Profiling in Biliary Tract Infections: A Comparative Study With Clinical Stratification**

Jianmin Ren^1†^, Ziling Lan^1†^, Cheng Wang^2^, Jinnuo Zhu^1^, Mei Li^1^, Jianfen Xu^2^, Yiyang Lu^2^, Jianfei Tu^2^, Xiaoyao Zhang^2^, Lidija Boskovic^3^, Jiansheng Huang^1,2*^, Xiaolei Hu^1,2*^

1. Graduate Joint Training Base of Zhejiang Chinese Medical University (Lishui Joint Training Base - Lishui Central Hospital), Lishui, China
2. Department of Clinical Laboratory, The Fifth Affiliated Hospital of Wenzhou Medical University, Lishui, Zhejiang, China
3. Department of Microbiology and Genetics, Clinical Hospital Center Zvezdara, Belgrade, Serbia.

^†^These authors contributed equally to this work.

* Corresponding author.

1. mail address: sherryhu830416@126.com(Xiaolei Hu); Huangxvy@163.com (Jiansheng Huang).

Table S1. Distribution of major microorganisms detected by mNGS in bile samples, stratified by clinical characteristics

| \| microorganisms \| \| --- \| | **Total**  **(n)** |  | **Gender (n)** | | | |  | **Age (n)** | | | |  | **Severity (n)** | | | |  | **Infection site (n)** | | | |  | **Tumor type (n)** | | | |  | **Stone type (n)** | | | |
| --- | --- | --- | --- | --- | --- | --- | --- | --- | --- | --- | --- | --- | --- | --- | --- | --- | --- | --- | --- | --- | --- | --- | --- | --- | --- | --- | --- | --- | --- | --- | --- | --- |
|  |  |  | **M** | **F** | ***P*** | ***q*** |  | **E** | **Y** | ***P*** | ***q*** |  | **M-S** | **M** | ***P*** | ***q*** |  | **B** | **G** | ***P*** | ***q*** |  | **T** | **non-T** | ***P*** | ***q*** |  | **S** | **Non-S** | ***P*** | ***q*** |
| Total samples | 99 |  | 54 | 45 |  |  |  | 52 | 47 |  |  |  | 48 | 51 |  |  |  | 42 | 57 |  |  |  | 35 | 64 |  |  |  | 50 | 49 |  |  |
| **Gram-positive Bacteria** | 119 |  | 70 | 32 |  |  |  | 77 | 75 |  |  |  | 112 | 80 |  |  |  | 96 | 51 |  |  |  | 108 | 87 |  |  |  | 71 | 111 |  |  |
| *Streptococcus* | 31 |  | 16 | 9 | 0.479 | 0.670 |  | 23 | 18 | 0.373 | 0.610 |  | 28 | 19 | 0.06 | 0.210 |  | 25 | 11 | 0.019 | 0.133 |  | 29 | 19 | 0.012 | 0.168 |  | 12 | 30 | 0.002 | 0.056 |
| *Enterococcus* | 24 |  | 17 | 4 | 0.007 | 0.045 |  | 17 | 11 | 0.256 | 0.523 |  | 21 | 15 | 0.287 | 0.402 |  | 19 | 9 | 0.029 | 0.152 |  | 21 | 22 | 0.847 | 0.931 |  | 17 | 22 | 0.441 | 0.617 |
| *Staphylococcus* | 13 |  | 7 | 5 | 0.758 | 0.795 |  | 7 | 8 | 0.788 | 0.855 |  | 8 | 10 | 0.62 | 0.620 |  | 5 | 10 | 0.006 | 0.045 |  | 7 | 14 | 0.343 | 0.600 |  | 11 | 10 | 0.814 | 0.814 |
| *Actinomyces* | 12 |  | 8 | 2 | 0.191 | 0.401 |  | 8 | 5 | 0.567 | 0.738 |  | 10 | 9 | 1.000 | 1.000 |  | 9 | 6 | 0.263 | 0.460 |  | 10 | 10 | 0.648 | 0.864 |  | 8 | 11 | 0.629 | 0.734 |
| *Parvimonas* | 10 |  | 6 | 3 | 0.726 | 0.795 |  | 5 | 7 | 0.552 | 0.738 |  | 10 | 5 | 0.269 | 0.402 |  | 8 | 3 | 0.120 | 0.280 |  | 9 | 6 | 0.289 | 0.600 |  | 5 | 9 | 0.401 | 0.617 |
| *Granulicatella* | 8 |  | 2 | 3 | 0.378 | 0.588 |  | 4 | 5 | 0.734 | 0.822 |  | 7 | 5 | 0.764 | 0.764 |  | 6 | 2 | 0.147 | 0.309 |  | 7 | 4 | 0.366 | 0.600 |  | 3 | 7 | 0.332 | 0.617 |
| *Rothia* | 7 |  | 3 | 1 | 1.000 | 0.920 |  | 3 | 4 | 0.708 | 0.822 |  | 6 | 3 | 0.497 | 0.580 |  | 5 | 2 | 0.260 | 0.460 |  | 6 | 4 | 0.52 | 0.728 |  | 3 | 6 | 0.497 | 0.662 |
| *Abiotrophia* | 5 |  | 2 | 1 | 1.000 | 0.920 |  | 2 | 3 | 0.663 | 0.822 |  | 5 | 3 | 0.716 | 0.764 |  | 4 | 1 | 0.38 | 0.532 |  | 4 | 3 | 0.716 | 0.836 |  | 2 | 5 | 0.441 | 0.617 |
| *Gemella* | 3 |  | 1 | 1 | 1.000 | 0.920 |  | 2 | 2 | 1.000 | 1.000 |  | 3 | 3 | 1.000 | 1.000 |  | 3 | 1 | 0.624 | 0.728 |  | 3 | 2 | 1.000 | 1.000 |  | 2 | 3 | 1.000 | 1.000 |
| *Cutibacterium* | 3 |  | 0 | 3 | 0.078 | 0.273 |  | 3 | 1 | 0.623 | 0.812 |  | 1 | 4 | 0.363 | 0.508 |  | 2 | 3 | 0.655 | 0.728 |  | 1 | 4 | 0.386 | 0.600 |  | 3 | 2 | 0.680 | 0.756 |
| Other Gram-positive | 13 |  | 8 | 0 | 0.020 | 0.093 |  | 3 | 11 | 0.008 | 0.088 |  | 13 | 4 | 0.057 | 0.210 |  | 10 | 3 | 0.079 | 0.221 |  | 11 | 9 | 0.487 | 0.728 |  | 5 | 12 | 0.115 | 0.322 |
| **Gram-negative Bacteria** | 163 |  | 89 | 30 |  |  |  | 94 | 62 |  |  |  | 133 | 83 |  |  |  | 116 | 67 |  |  |  | 122 | 126 |  |  |  | 100 | 152 |  |  |
| *Klebsiella* | 26 |  | 15 | 7 | 0.330 | 0.578 |  | 18 | 12 | 0.289 | 0.523 |  | 22 | 15 | 0.199 | 0.348 |  | 19 | 14 | 0.228 | 0.425 |  | 20 | 23 | 0.858 | 0.931 |  | 16 | 24 | 0.240 | 0.560 |
| *Escherichia* | 23 |  | 11 | 5 | 0.418 | 0.627 |  | 16 | 8 | 0.092 | 0.304 |  | 18 | 14 | 0.534 | 0.623 |  | 18 | 10 | 0.09 | 0.231 |  | 16 | 21 | 0.713 | 0.836 |  | 16 | 21 | 0.560 | 0.662 |
| *Enterobacter* | 17 |  | 10 | 1 | 0.026 | 0.118 |  | 13 | 3 | 0.018 | 0.132 |  | 15 | 7 | 0.106 | 0.247 |  | 12 | 5 | 0.082 | 0.231 |  | 13 | 16 | 1.000 | 1.000 |  | 10 | 16 | 0.388 | 0.617 |
| *Veillonella* | 12 |  | 5 | 3 | 1.000 | 0.920 |  | 7 | 6 | 1.000 | 1.000 |  | 10 | 6 | 0.429 | 0.550 |  | 9 | 4 | 0.153 | 0.309 |  | 10 | 8 | 0.463 | 0.728 |  | 5 | 11 | 0.193 | 0.54 |
| *Campylobacter* | 11 |  | 7 | 1 | 0.138 | 0.322 |  | 7 | 3 | 0.332 | 0.575 |  | 9 | 4 | 0.249 | 0.402 |  | 7 | 6 | 0.771 | 0.770 |  | 8 | 12 | 0.818 | 0.931 |  | 9 | 10 | 1.000 | 1.000 |
| *Haemophilus* | 11 |  | 4 | 3 | 1.000 | 0.920 |  | 5 | 5 | 1.000 | 1.000 |  | 7 | 7 | 1.000 | 1.000 |  | 7 | 3 | 0.203 | 0.395 |  | 8 | 7 | 0.593 | 0.792 |  | 5 | 10 | 0.284 | 0.597 |
| *Bacteroides* | 11 |  | 4 | 1 | 0.649 | 0.759 |  | 6 | 2 | 0.280 | 0.523 |  | 9 | 5 | 0.406 | 0.550 |  | 8 | 6 | 0.581 | 0.677 |  | 8 | 10 | 1.000 | 1.000 |  | 8 | 10 | 0.810 | 0.814 |
| *Fusobacterium* | 9 |  | 3 | 1 | 1.000 | 0.920 |  | 3 | 4 | 0.708 | 0.822 |  | 7 | 4 | 0.537 | 0.623 |  | 6 | 4 | 0.528 | 0.637 |  | 7 | 7 | 1.000 | 1.000 |  | 5 | 8 | 0.577 | 0.673 |
| *Clostridium* | 8 |  | 4 | 1 | 0.649 | 0.759 |  | 3 | 2 | 1.000 | 1.000 |  | 6 | 4 | 0.744 | 0.764 |  | 5 | 3 | 0.488 | 0.621 |  | 5 | 8 | 0.774 | 0.931 |  | 6 | 7 | 1.000 | 1.000 |
| *Proteus* | 6 |  | 1 | 2 | 0.560 | 0.712 |  | 3 | 2 | 1.000 | 1.000 |  | 4 | 3 | 1.000 | 1.000 |  | 4 | 2 | 0.431 | 0.569 |  | 4 | 5 | 1.000 | 1.000 |  | 4 | 6 | 0.747 | 0.814 |
| Other Gram-negative | 30 |  | 25 | 5 | 0.019 | 0.093 |  | 13 | 16 | 0.275 | 0.523 |  | 26 | 14 | 0.096 | 0.247 |  | 21 | 10 | 0.052 | 0.182 |  | 23 | 25 | 0.589 | 0.792 |  | 16 | 29 | 0.08 | 0.299 |
| **Fungi & Viruses** | 57 |  | 28 | 14 |  |  |  | 31 | 16 |  |  |  | 40 | 23 |  |  |  | 36 | 24 |  |  |  | 42 | 42 |  |  |  | 32 | 51 |  |  |
| **Fungi** |  |  |  |  |  |  |  |  |  |  |  |  |  |  |  |  |  |  |  |  |  |  |  |  |  |  |  |  |  |  |  |
| *Candida* | 14 |  | 6 | 5 | 0.744 | 0.795 |  | 8 | 4 | 0.363 | 0.610 |  | 10 | 8 | 0.804 | 0.804 |  | 10 | 5 | 0.164 | 0.319 |  | 11 | 10 | 0.342 | 0.600 |  | 7 | 13 | 0.334 | 0.617 |
| Other fungi | 4 |  | 2 | 1 | 1.000 | 0.920 |  | 3 | 1 | 0.623 | 0.812 |  | 3 | 1 | 0.621 | 0.62 |  | 1 | 4 | 0.163 | 0.319 |  | 2 | 4 | 0.691 | 0.836 |  | 4 | 2 | 0.439 | 0.617 |
| **Viruses** |  |  |  |  |  |  |  |  |  |  |  |  |  |  |  |  |  |  |  |  |  |  |  |  |  |  |  |  |  |  |  |
| *Cytomegalovirus* | 17 |  | 7 | 4 | 1.000 | 0.920 |  | 11 | 3 | 0.036 | 0.049 |  | 12 | 5 | 0.108 | 0.247 |  | 11 | 5 | 0.095 | 0.231 |  | 13 | 10 | 0.238 | 0.600 |  | 7 | 16 | 0.080 | 0.299 |
| *Torque teno virus* | 13 |  | 9 | 2 | 0.196 | 0.401 |  | 8 | 3 | 0.206 | 0.453 |  | 10 | 4 | 0.169 | 0.315 |  | 8 | 5 | 0.396 | 0.554 |  | 10 | 10 | 0.638 | 0.864 |  | 7 | 12 | 0.467 | 0.654 |
| *Hepatitis B virus (HBV)* | 6 |  | 2 | 2 | 1.000 | 0.920 |  | 1 | 4 | 0.183 | 0.453 |  | 2 | 5 | 0.257 | 0.402 |  | 3 | 4 | 0.701 | 0.770 |  | 3 | 6 | 0.730 | 0.836 |  | 5 | 5 | 1.0000 | 1.0000 |
| Other Viruses | 3 |  | 2 | 1 | 1.000 | 0.920 |  | 0 | 1 | 0.467 | 0.684 |  | 3 | 0 | 0.242 | 0.402 |  | 3 | 1 | 0.338 | 0.497 |  | 3 | 2 | 0.657 | 0.864 |  | 2 | 3 | 1.0000 | 1.0000 |

Note: M:Male;F:Female;*P*:*P*-value;*q*:*q*-value;E:Elderly;Y:Younger;M-S:Moderate–Severe;M:Mild;B:Bile Duct;G:Gallbladder;T:With Tumor;non-T:Without Tumor;S:With Stone;non-S:Without Stone.

Statistical analyses were performed using Fisher’s exact test for intergroup comparisons of positive rates. A significance level of α = 0.05 was applied. The Benjamini–Hochberg procedure was used to adjust P-values for multiple testing, controlling the false discovery rate (FDR); adjusted *q*-values are reported. An asterisk (*) denotes statistical significance (*q* < 0.05). These subgroup comparisons are exploratory and are presented to describe detection patterns rather than to establish independent epidemiologic associations.

Elderly: ≥ 60 years; Younger: < 60 years.

Group definitions: Moderate–Severe (TG18 Grades II–III), Mild (TG18 Grade I); Infection Site: Bile Duct vs. Gallbladder; With/Without Tumor; With/Without Stone.

Bacterial taxa are summarized at the genus level for statistical analysis, while fungi, viruses, and parasites are reported at the species level.
